# Modeling the Natural History and Detection of Lung Cancer Based on Smoking Behavior

**DOI:** 10.1371/journal.pone.0093430

**Published:** 2014-04-04

**Authors:** Xing Chen, Millennia Foy, Marek Kimmel, Olga Y. Gorlova

**Affiliations:** 1 Department of Biomedical Engineering, Key Laboratory of Biomedical Engineering of Ministry of Education of China, Zhejiang University, Hangzhou, Zhejiang, China; 2 Department of Epidemiology, The University of Texas MD Anderson Cancer Center, Houston, Texas, United States of America; 3 Brown Foundation Institute of Molecular Medicine, The University of Texas Health Science Center at Houston, Houston, Texas, United States of America; 4 Departments of Statistics and Bioengineering, Rice University, Houston, Texas, United States of America; 5 Department of Community and Family Medicine, Geisel School of Medicine at Dartmouth College, Lebanon, New Hampshire, United States of America; University of California Irvine, United States of America

## Abstract

In this study, we developed a method for modeling the progression and detection of lung cancer based on the smoking behavior at an individual level. The model allows obtaining the characteristics of lung cancer in a population at the time of diagnosis. Lung cancer data from Surveillance, Epidemiology and End Results (SEER) database collected between 2004 and 2008 were used to fit the lung cancer progression and detection model. The fitted model combined with a smoking based carcinogenesis model was used to predict the distribution of age, gender, tumor size, disease stage and smoking status at diagnosis and the results were validated against independent data from the SEER database collected from 1988 to 1999. The model accurately predicted the gender distribution and median age of LC patients of diagnosis, and reasonably predicted the joint tumor size and disease stage distribution.

## Introduction

Lung cancer is one of the most deadly diseases worldwide, largely because most patients present with advanced-stage disease at the time of diagnosis [1], [2]. Most patients have clinical stage III or IV disease when they first notice symptoms and seek medical attention, which results in a poor prognosis. Age, gender, smoking status, tumor size, and disease stage at the time of diagnosis are highly related to the prognosis of patients with lung cancer [3], [4], [5]. In this article, we use mathematical methods to disentangle the tumorigenesis and detection processes. The goal of this model is to trace the timeline of an individual from his/her birth to the time of lung cancer initiation, progression, detection, and death. Thus, we combined models of carcinogenesis (cancer development until the first malignant cell), tumor progression (growth and metastasis), and detection, to construct a framework for modeling lung cancer at an individual level. Using this framework, we could infer characteristics that cannot be observed in clinical practice, including age of the patient when the primary tumor and nodal and distant metastases are formed. We were also able to evaluate characteristics that can only be partially observed, such as the tumor growth rate, and closely reconstruct characteristics that can be observed clinically, notably, tumor size and disease stage at the time of diagnosis. This procedure may be useful for better understanding the formation of the current lung cancer patient population and characterization of the future lung cancer trends with changes in smoking behavior and detection methods.

## Materials and Methods

### 1. Lung cancer patients identified in the Surveillance, Epidemiology and End Results (SEER) database

Age, sex, disease stage, and tumor size for lung cancer patients who were diagnosed between 1973 and 2008 are available in the SEER database [6]. For patients diagnosed from 1988 to 1999, we used information on tumor size, with staging determined according to SEER extent of disease codes, which categorize tumors as localized, regional, and distant. For patients diagnosed from 2004 to 2008, the staging system developed by the American Joint Committee on Cancer was used to obtain tumor size and tumor node metastasis (TNM) disease stage information. Tumor size was measured as the maximum diameter, and we calculated the volume according to an assumption that a tumor grows as a sphere. We re-categorized the tumors into groups from 0 to 20 cm (with 1-cm increments) according to their maximum diameter.

### 2. Carcinogenesis modeling

For the carcinogenesis model, we used the two-stage clonal expansion (TSCE) model developed by Moolgavkar and Venzon [7] to calculate the age of the patient at tumor initiation. This model leads to an explicit formula for the distribution of the total duration, *T*, of the first two stages in the carcinogenesis process (the transitions from normal to initiated cell and initiated to malignant cell), which encompasses the time from the birth of an individual to the onset of malignancy [8]. A smoking-based modification of the TSCE model relating smoking intensity measured in packs per day (ppd) to the parameters of the TSCE model through response functions (with the parameters *ν*_0_, *α*_0_, *γ*_0_, *a*_1_, and *a*_2_, as listed in File S1) was chosen. Smoking duration was incorporated to produce a more specific Survival function of the age at tumor initiation for individual never, current and former smokers.[9], [10], [11].

The smoking history generator (SHG, version 5.2.1) [12]from the Cancer Intervention and Surveillance Modeling Network (CISNET), which produced smoking duration and intensity data for individuals, was incorporated into the smoking-based TSCE model by using the parameters listed in Table S1. Parameters for males and females were estimated using the data from Cancer Prevention Study I (CPS-I) and from Nurses' Health Study (NHS), respectively. The SHG was also used to generate an age of death due to causes other than lung cancer.

### 3. Tumor growth and metastasis modeling

For the tumor growth and metastasis models, we assumed that the hazard of tumor progression is based on the activity of the tumor cells, and tumor cells detach from the primary tumor and transfer to another part of the body, leading to metastases [13]

#### 3.1. Assumptions

The following assumptions were made in modeling tumor growth and metastasis:

1. The primary tumor grows from a single cell, with an assumed volume of 1×10^−9^ cm^3^ [14]. The growth rate λ, which is related to the tumor doubling time by the expression 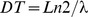, is determined at the time of tumor initiation and is assumed to remain the same over time.
2. The growth rate follows a gamma distribution, with shape and scale parameters θ and K.
3. All metastases are derived from the primary tumor, which means cells detach from the primary tumor at the rate ξ and are transferred and deposited at the rate μ at a new metastasis site [13]. We don't consider secondary metastasis from existing metastasis.
4. The activity of the tumor cell is related to how fast the tumor grows and how easily the cells detach. Specifically, the faster the primary tumor grows, the easier it is for the cells to detach. We define the tumor cell's activity α, to which the growth rate λ is proportional, λ = ε1×α (where ε1 is a constant). The cell-detachment rate, β, is also proportional to α, β = ε2×α (where ε2 is another constant). Thus, 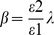, where 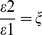 is a parameter representing the relationship between β and λ. If the tumor with volume S grows exponentially, 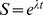, the total number of detached cells before time *τ*_0_ is 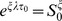; we assume 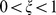, the interpretation of which is that cells always detach from the primary tumor but not all tumor cells will detach.
5. The detached cells will be transferred and deposited at new locations. The aggregate rate of transfer and deposition is μ. μ could be a constant parameter or a functional parameter determined by a biological process, such as the rate of synthesis of proteins that help transfer the tumor cells across the blood vessel wall [13].
6. Metastases are defined as either nodal or distant. We assume a different rate μ (μ_n_ and μ_m_) for each type of metastasis, μ_n_ for nodal and μ_m_ for distant metastasis. We also assume that the detached cells can move to nodal sites at least as easily as to distant sites (μ_n_≥μ_m_).
7. The hazard function for metastasis (nodal or distant) is related to the number of tumor cells that have detached from the primary tumor and have been successfully transferred and deposited at nodal or distant locations. Assuming exponential growth, the hazard functions for nodal and distant metastases are 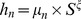 and 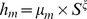, respectively. The cumulative distribution functions (c.d.f.) are defined below:

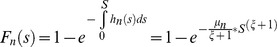





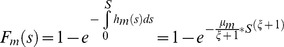
with the tail functions (or survival functions) 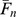, 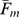. If 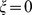, we assume no cells detach from the primary tumor, 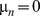 and 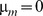.

1. Assumption μ_n_≥μ_m_ implies that 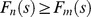, where F_n_(s) and F_m_(s) are corresponding cumulative distribution functions defined above. Primary tumor sizes at the time of initiation for nodal and distant metastases, respectively, are denoted 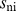
and 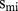.
2. We assume that the cell's activity changes after detachment from the tumor, transfer and deposition at the metastatic site. Cell at the nodal and distant metastatic sites grow three (3λ) or four (4λ) times faster than the primary tumor [13], [15], [16], correspondingly.

The primary tumor size is calculated using the tumor growth model by giving the growing time *t*, with a constant growth rate λ. Thus, we rewrite 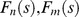 to 
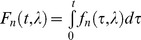
 and 
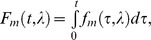
 where 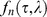 and 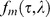 are the probability density functions (p.d.f.) of time that nodal and distant metastases happened in a group of patients with the same tumor growth rate λ. Then,
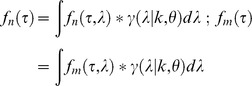
where 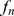 and 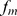 are the p.d.f. of time that nodal and distant metastases occurred in patients with tumor growth rate having a Gamma distribution, and 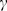 is the Gamma distribution function with parameters 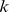 and 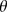. Then, 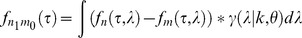
where 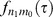 is the p.d.f. of time for patients who had only nodal metastases (without distant metastases) in the whole time period.

### 4. Cancer detection modeling

To model cancer detection, we introduced a competing process of detecting the disease through the primary tumor or nodal or distant metastases, adapting the framework developed by Kimmel and Flehinger. [17]

#### 4.1. Assumptions

The following assumptions were made in modeling cancer detection:

1. The detection of cancer is based on the detection method used. The hazard of detection has a linear relationship with tumor size. It also depends on the reasons (e.g., symptoms or results of a screening test) that prompted the patient to seek medical attention. For details, see equations (*) for 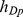, 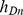, and 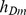 further on.
2. The detection of cancer is considered a competing process of detecting the primary tumor or nodal or distant metastases. The specific, mode-dependent hazard functions for the detection through the primary tumor and nodal and distant metastases are denoted as 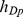, 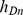, and 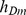 respectively (*). Then the c.d.f. of the detection of primary tumors and nodal and distant metastases are denoted as D_p_(s), D_n_(s), and D_m_(s), (**) with tail functions 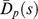, 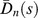,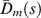, respectively (for details see equations (**)).
3. We also define primary tumor-size dependent hazard functions for detection (irrespective of the mode of detection, whether through the primary tumor, nodal metastases, or distant metastases), z_00_(s), z_10_(s), z_01_(s), and z_11_(s), where z_00_(s) is the hazard function for detecting, at size s, a cancer with no detectable metastases; z_10_(s) is the hazard of detecting, through whatever means, a cancer with the primary tumor of size s and with detectable nodal but not distant metastases; likewise, z_01_(s) is the hazard function for detecting, at size s, a cancer with detectable distant but not nodal metastases; and z_11_(s) is the hazard function for detecting, at size s, a cancer with detectable nodal and distant metastases. Associated with these hazard functions are the c.d.f. Z_nm_(s) before they reach size S, with tails 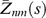, where n, m = 0,1.
4. The observable variables in the study of size-dependent metastases are sizes S of the primary tumor at detection and the indicators N and M, where N, M = 1/0 if nodal or distant metastases are present/absent.

The relationship between assumptions (2) and (3) for the detection model is shown in the following functions. 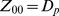





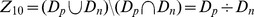





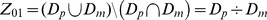





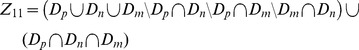
To give the explicit expressions for 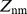, the detailed expressions of 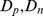 and 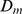
are required. According to the assumptions of metastases model, we know that: 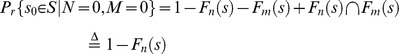





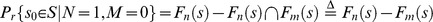





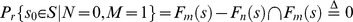





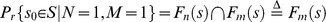
Where 0<S_0_≤S, is the size of the primary tumor that was not observed and Δ is considered the assumption that 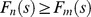. 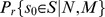 represented the probability of a primary tumor with (1) or without (0) nodal (N) or distant (M) metastasis.

The joint density/ probability functions 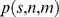 of random variable S, N and M are presented below.
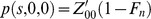





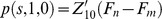





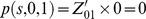





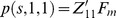
Where 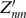 are probability density functions for detection.

The tumor-size dependent probability that nodal and distant metastases are present at diagnosis, 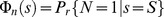 and 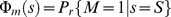, respectively, where
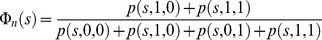





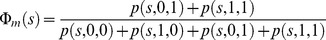
Substitute 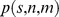 with 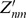,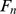, and 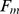 we obtain:



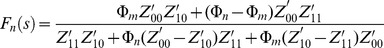





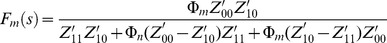



### 5. Estimation

Methods provided by Kimmel and Flehinger [17] could be used to estimate 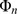, 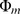, and 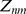 non-parametrically. We can also estimate 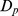,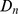,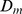,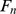, and 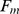 parametrically once the parametric tumor-growth and detection models are determined. Below we provide an example.

Assuming that a tumor grows exponentially with a growth rate λ, and the metastases model described above, we have 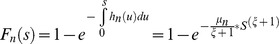





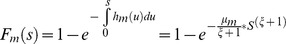
Assuming that the hazard of tumor detection depends linearly on the size of the tumor, denoting the efficiency of the detection by tumor size as α and stage-dependent offset parameters as w_0_, w_1_ and w_2_, we obtain



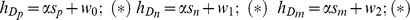
 Correspondingly 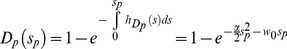
(**)




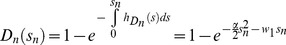
(**)




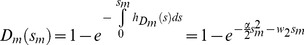
(**)are c.d.f. of detection by size 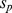
or 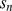 or 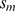
of the primary tumor and nodal and distant metastases. The primary tumor size when the nodal and distant metastases arise is denoted as 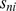,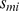. We can then rewrite 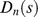, 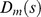 as
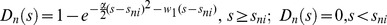





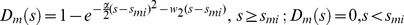
where 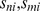 are distributed with c.d.f. 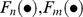, respectively, and 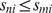.

#### 5.1. Simulation-based estimation

The tumor growth and metastasis model includes nine parameters (ξ, 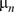, 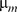, 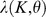, α, W_0_, W_1_, W_2_). The joint likelihood function is difficult to maximize directly. However, the tumor-growth, metastasis, and detection models can be estimated separately, once multiple data points for tumor size and disease stage are available. Another method is to derive the least-squares function: 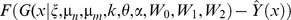, where 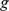 is the simulated joint distribution of tumor size and stage and 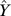 is the observed joint distribution, based on these parameters, and apply the Nelder-Mead method [18], [19], [20] to achieve the best fitted parameters in the model. We used the second approach because we did not have the multiple tumor-size measurements for individuals to estimate the models separately.



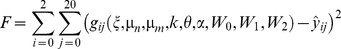
is the least square function where *i* is the stage status defined as local (*i* = 0, no nodal or distant metastasis N0M0), nodal (*i* = 1, nodal metastases but no distant metastases, N1M0), or distant (*i* = 2, M1), *j* is the number of tumor size group; 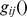 is the simulated percentage of lung cancer with tumor in the size range of *j* group and *i* stage among all detected lung cancer; 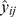 is the observed percentage of lung cancer with tumor in the size range of j group and i stage among all detected lung cancer. Detailed simulation procedure was in File S1.

We estimated the nine parameters (*ξ*, 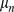, 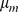, 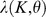, η, *W*_0_, *W*_1_, *W*_2_) using the TNM staging data in the SEER database from 2004 to 2008 for model fitting. Since SHG is using year 2000 as a cut-off point for the vital status observation, the joint distributions of tumor size and disease stage from 1995 to 1999 were chosen as the output of the simulation. The results were validated against independent data from the SEER database collected from 1988 to 1999. These years were chosen as closest possible to 2004–2008 periods.

To simulate the LC population, we firstly used the smoking history generator (SHG) to generate the underlying population. We assumed that the number of persons before year 1890 was zero and at the year of 1890 there were 2877000 new born babies (the number of live births in each year was shown in the Figure S1). We provided the year of birth (say 1890) and gender (half and half) to SHG as inputs and repeated the SHG for 287700 times. Then we got these persons' basic information, including the year of death (converted from the age of death, A_d_, generated by SHG) and their smoking history information. We then applied our simulation strategy (described in the File S1 at section 2.1 simulation process) to get LC candidates and the information of their tumor progression. For the next year (say 1891), we added new born babies to the underlying population and removed the persons that were dead in the previous year (say 1890) from the population, whenever she or he was LCs or “normal” persons. Thus, we had underlying population, which would be approaching the real U.S. population (figure S2), and the LC candidate population, which were considered as an unperturbed (existing before detection) LC population. Assuming that no LC-related death occurred before detection, the yearly LC population would be achieved by applying the detection model to the unperturbed LC population.

## Results

**Figure 1:**
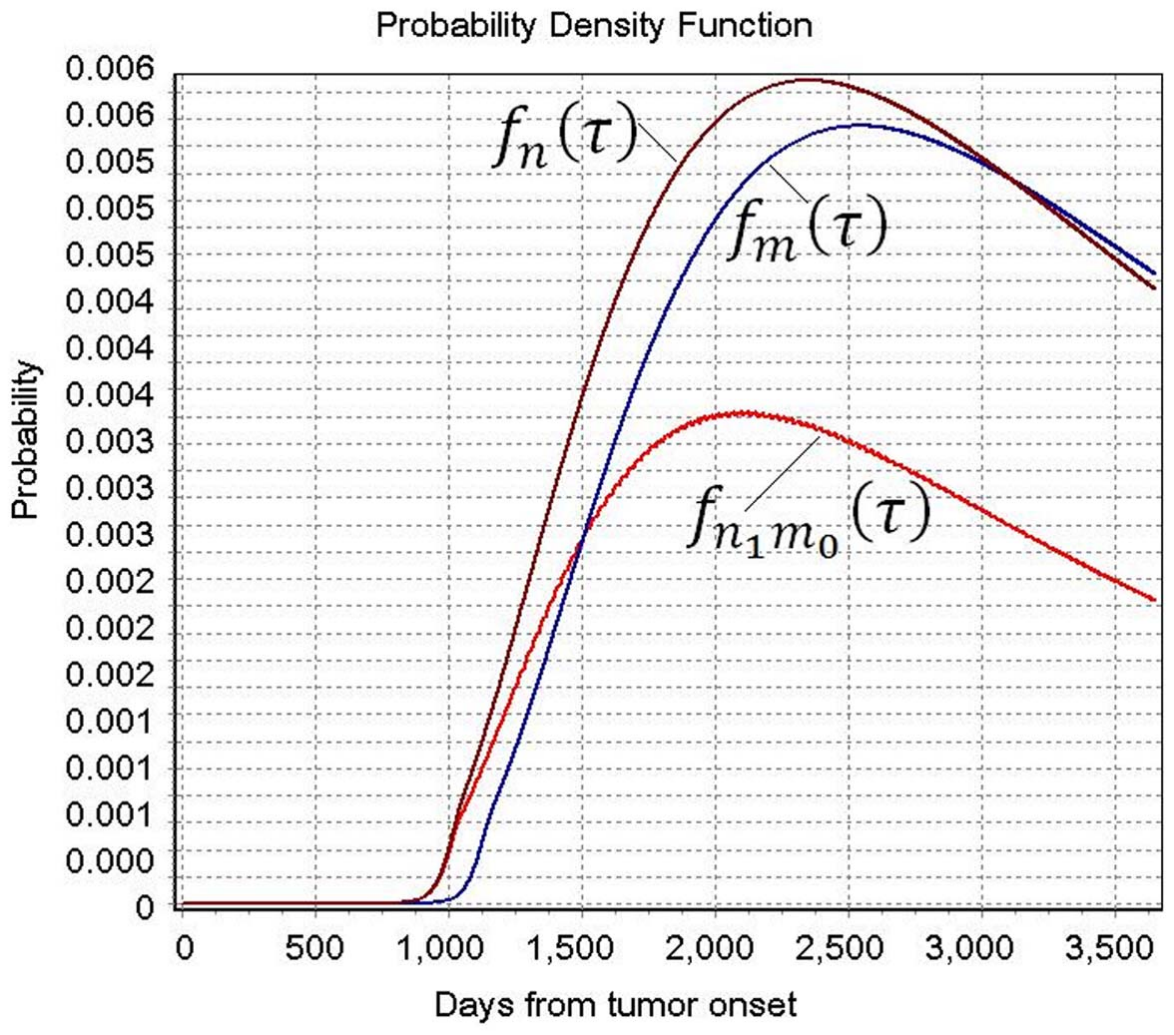
Probability density functions of nodal and distant metastases from the time of tumor onset, using the estimated parameters ξ = 0.01, 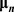
 **= 8.05×10^−9^,** 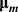 **= 2.78×10^−9^, K = 3.80 and** 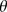 **= 1.15.**

Figure 1 shows the probabilities of nodal metastases and distant metastases by the time from the tumor onset. The estimated parameters ξ, 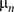, 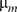, *k* and 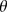 in Table 1, which gives the estimates of the model parameters, were used to draw 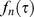, 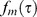 and 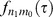. These probability density functions showed that the probability of nodal and distant metastasis began to fast increase at 2.5 years (about 900 days) and 3 years (about 1100 days) from the time of tumor onset, respectively. It reached the highest at 6.4 years (about 2350 days) and 6.8 years (about 2500 days) from the time of tumor onset.

**Table 1:** The estimates of model parameters, with asymptotic confidence intervals.

Parameter	Description	Estimate	95% CI
ξ	Detachment rate	0.01	[0.008, 0.011]
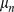	Transfer and deposition rate of cells to nodal metastases	8.05×10^−9^	[7.80×10^−9^, 8.21×10^−9^]
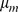	Transfer and deposition rate of cells to distant metastases	2.78×10^−9^	[2.15×10^−9^, 3.34×10^−9^]
* 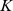	Shape parameter of gamma distribution of tumor growth rate	3.80	[3.77, 3.82]
* 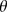	Scale parameter of gamma distribution of tumor growth rate	1.15	[1.12, 1.19]
η	Efficiency of the detection by tumor size	1.0×10^−4^	[1.0×10^−5^, 1.0×10^−3^]
W_0_	Offset parameter for detection by N0M0 stage symptoms	0.065	[0.056, 0.075]
W_1_	Offset parameter for detection by N1M0 stage symptoms	1.50×10^3^	[1.30×10^3^, 1.80×10^3^]
W_2_	Offset parameter for detection by M1 Stage symptoms	7.00×10^4^	[6.50×10^3^, 8.00×10^5^]

*Assuming exponential tumor growth and the estimates of K and θ, the average tumor growth rate E(λ) corresponds to a doubling time of 55 to 60 days.

### 1. Model Fitting

**Figure 2:**
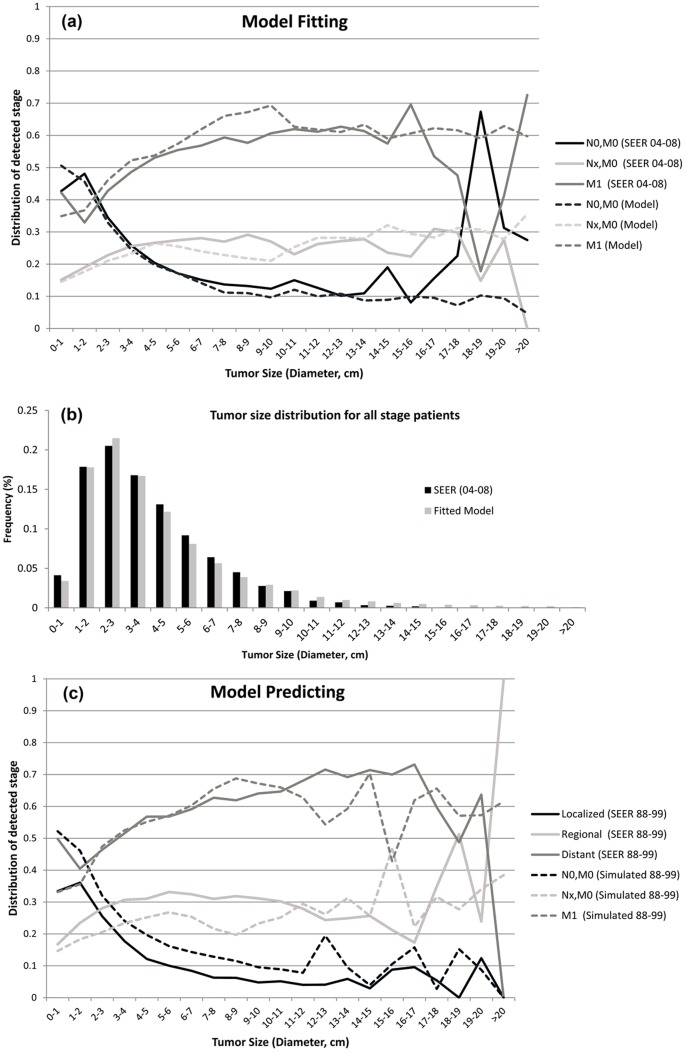
Comparison of the model fit in the period of 1995–1999 to the data of SEER 2004–2008. (a) the stage distribution conditional on tumor size and (b) the tumor size distribution; (c) Comparison of the predictive model 1988–1999 and SEER 1988–1999, where the data is summarized as stage distribution conditional on tumor size.

Figure 2 compares the characteristics of the population for the years 1995–1999 generated by the fitted model to the SEER data (2004–2008). For tumors smaller than 10 cm in diameter, the proportion of N0M0-stage disease (no nodal or distant metastases) more closely reproduces the SEER data (2004–2008). The proportions of NxM0- and M1-stage disease are not reproduced as accurately as the proportions of N0M0-stage disease, especially when the tumors are larger than 5 cm in diameter. For tumors smaller than 1 cm, the model predicted that about 50% and 35% would be staged as N0M0 and M1, respectively, whereas the actual percentages were 42% and 42% respectively.

### 2. Predicting Clinically Observable Characteristics

The fitted model was also validated by predicting the characteristics of lung cancer patient population in United States between 1988 and 1999. The model predicts both the gender distribution among LC patients and median age that are quite close to the 1988–1999 SEER data (Table 2).

**Table 2:** Comparison of the lung cancer patient population predicted by our model (1988 to 1999) with data from the SEER database (from 1988 to 1999 and from 2004 to 2008).

Characteristics	Prediction 1988 to 1999 (N = 1,434,024)	SEER 1988 to 1999 (N = 184,952)	SEER 2004 to 2008 (N = 84,422)
Sex, n (%)			
Male	833,754 (58.1)	108,205 (58.5)	44,228 (52.4)
Female	600,270 (41.9)	76,747 (41.5)	40,194 (47.6)
Age			
Mean(SD)	67.31(14.26)	68.06(10.98)	69.75 (11.57)
Median	69	69	71
*Stage, n (%)			
N0,M0	380,017 (26.5)	31,432 (17.0)(19.3)£	20,863 (24.7)(28.1)£
Nx,M0	321,221 (22.4)	46,934 (25.4)(28.8)£	17,889 (21.2)(24.1)£
M1	732,786 (51.1)	84,519 (45.7)(51.9)£	35,357 (41.9)(47.7)£
Missing stage	0 (0)	22,067 (11.9)	10,313 (12.2)
Tumor Size, cm (Diameter)			
Mean	4.57	4.30	4.21
Median	3.69	3.80	3.50
Std. deviation	3.21	3.03	3.11
Variance	10.33	9.20	9.65
Smoking Status, n (%)			
Never	185,885 (13.0)	**	-
Former	461,321 (32.2)	-	-
Current	786,818 (54.9)	-	-

*TNM staging being unavailable in SEER before 2004, we categorized tumors as localized, regional, and distant, for patients between 1988 and 1999.

**Smoking status is not reported in SEER.

£ Excluding missing stage.

**Figure 3:**
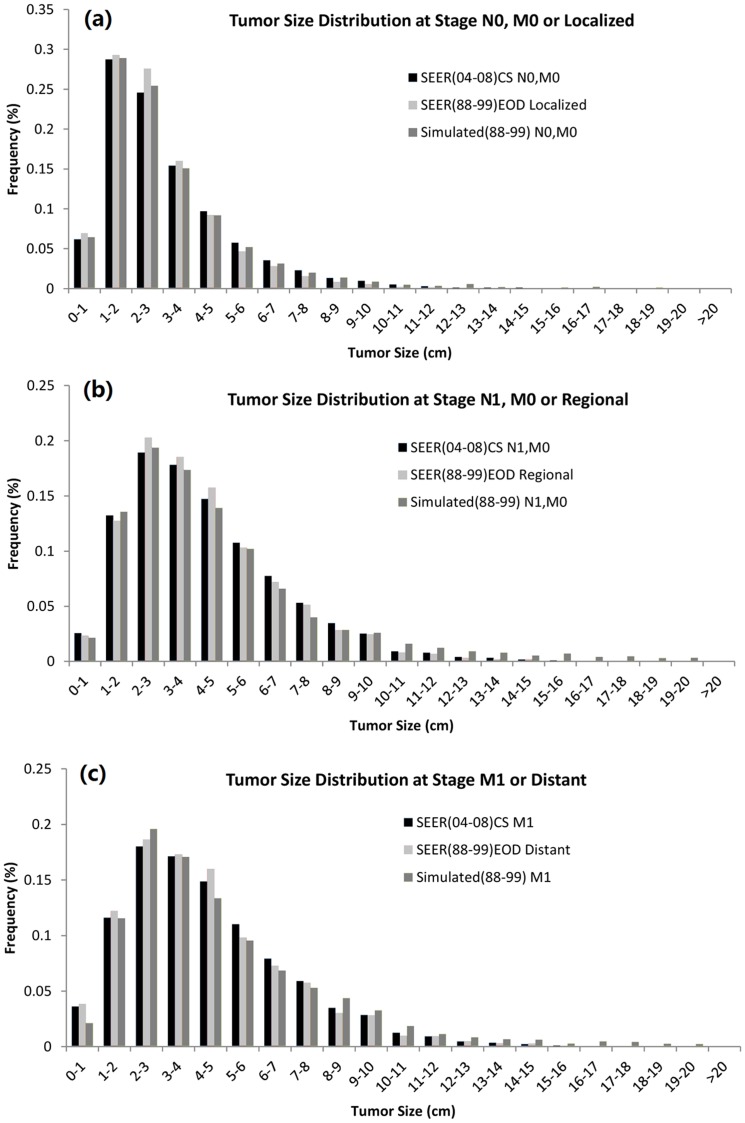
Tumor size distribution in predictive models, (a) Stage N0,M0 in SEER (2004–2008) and model (1988–1999), stage Localized by SEER standard in SEER (1988–1999), (b) Stage Nx,M0 (x≥1) in SEER (2004–2008) and model (1988–1999), stage Regional by SEER standard in SEER (1988–1999), (c) Stage M1 in SEER (2004–2008) and model (1988–1999), stage Distant by SEER standard in SEER (1988–1999).

Comparing the model prediction and the data, a smaller proportion of patients was diagnosed with localized disease than it was predicted (Figure 2c). One of the reasons may be the different staging definitions used. The predicted tumor size distributions were closer to the 2004–2008 SEER than to the 1988–1999 SEER data (Figure 3 (a–c)).

### 3. Predicting Clinically Unobservable Characteristics

Table 3 summarizes the predicted but not directly clinically observable characteristics of the detected tumors. The mean time from the tumor onset (when the first malignant cell appears) to nodal and distant metastases (when they are just formed and not yet observed) and diagnosis is about 4.77, 5.05, and 6.27 years, respectively. The average size of the primary tumors when nodal and distant metastases form was 0.09 cm^3^ and 0.24 cm^3^, respectively. The median age at the time of tumor onset is 63 and the average growth rate corresponds to the tumor-volume doubling time of about 60 days.

**Table 3:** Variables not directly observable for the detected tumors in the predicted lung cancer population (1988–1999).

Variables not directly observable	Mean (SD)	Median (IQR)
Time from the tumor onset, (years)		
To nodal metastasis	4.77 (2.70)	4.00 (3.00–6.00)
To distant metastasis	5.05 (2.86)	4.00 (3.00–6.00)
To diagnosis	6.27 (3.22)	5.00 (4.00–7.00)
Tumor volume (cm^3^) at metastasis		
S_n_	0.09 (8.4E-5)	0. 06 (0.03–0.13)
S_m_	0.24(2.8E-4)	0.16 (0.07–0.33)
The linear tumor dimension (cm) at metastasis		
D_n_	0.50 (1.7E-4)	0.49 (0.36–0.62)
D_m_	0.68 (2.8E-4)	0.67 (0.50–0.85)
*Yearly Growth Rate λ by stage		
N0M0	6.17 (2.91)	6.30 (3.70–8.15)
N1M0	5.61 (1.68)	5.43 (4.45–6.71)
M1	3.79 (1.55)	3.54 (2.64–4.81)
*Doubling time by stage, (days)		
N0M0	57.15 (43.83)	40.15 (31.02–68.41)
N1M0	49.40 (16.77)	46.52 (37.73–56.88)
M1	80.01 (38.66)	71.48 (52.65–95.69)

IQR, interquartile range; SD, standard deviation;* Here is the yearly growth rate and doubling time of primary tumor.

Table S2 shows the distribution of doubling time by tumor size and stage. In clinical practice, a primary tumor with distant metastases is more likely to be found with a smaller size than a primary with no or only nodal metastases. This leads to an observation that a primary tumor with distant metastases grows slower and remains smaller. This Table also demonstrates that faster growing tumors tend to be detected at larger sizes.

## Discussion

The parameters estimated from the joint distribution of tumor size and stage in the SEER database from 2004 to 2008 (Table 1) were applied to generate a lung cancer patient population from 1988 to 1999 and validated by comparison of the results to the data from the SEER database from 1988 to 1999. The model accurately predicts the gender distribution and the median age of lung cancer patients, and approximates the joint tumor size and disease stage distribution. The accurate prediction of gender distribution and age at diagnosis for 1988–1999 is largely owing to the accuracy of the smoking-based TSCE model and SHG. Because smoking behavior has changed significantly over the recent decades, the output is sensitive to the year at detection, which is why we are not able to reconstruct gender and age at diagnosis in the SEER data from 2004 to 2008 as accurately. This model overestimates the proportion of patients with tumors larger than 10 cm in diameter and underestimates the proportion of patients with tumors between 4 and 9 cm. These discrepancies are more obvious for the distributions of the primary tumor size at stages NxM0 and M1 than at N0M0. The reason might be that the detection interval is fixed to 1 year in our model, whereas patients may visit a doctor more frequently when symptoms appear. For tumors smaller than 1 cm, the model underestimated the proportion of patients with distant metastases.

We also used the fitted model to predict disease characteristics that are difficult or impossible to observe in clinical practice. According to the estimates of k and θ in Table 1, the average tumor growth rate, 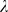, is about 4.4, which corresponds to a tumor-volume doubling time of approximately 55 to 65 days given the exponential tumor growth model. This growth rate is higher than what has been reported from screening studies [21], [22], [23], and thus the difference is not entirely unexpected [24]. Besides, introducing a time-dependent or size-dependent growth rate to the tumor growth model may improve the fit of the model to the data.

The hazards for detection once nodal or distant metastases are present are much larger than the hazards for detection when only the primary tumor is present (W_1_, W_2_>>W_0_), which reflects the reality of disease detection in clinical practice. The mean duration from tumor onset to detection was about 6 years in our model, which is consistent with other disease progression models [25]. Among the detected tumors, the average primary tumor size at the time of metastasis (nodal or distant) was less than 1 cm in diameter, which is considerably smaller than that implied by other predictions originating from screening data [17], [26], [27], [28]. Assumptions regarding detection used in the models led to this difference. In our model, the chances of detecting lung cancer increase after nodal and distant metastases occur, and the competitive detection model allows for the detection of the metastasized tumor. This detection model does not require either of the two extreme assumptions used in the previous studies [8], [9], [17]: (1) that the probability to detect cancer is unchanged when metastases are present, or (2) cancers are detected immediately when metastatic spread occurs. To reduce the complexity of the disease stage progression model, we did not consider the possibility of a secondary spread of the disease from nodal metastases. This may be the reason that the model did not as accurately reproduce the proportion of nodal and distant metastases as it did the proportion of localized tumors for tumor sizes larger than 5 cm.

Our framework combines a carcinogenesis model with a model of the natural history of tumor growth and progression, and a detection model, to predict features of a lung cancer patient population. This modular structure allows testing of different detection strategies. One limitation of this model is that we were not able to construct the overall likelihood function for the model in the analytical form, and the Nelder-Mead estimation procedure used to optimize the least square fit is time consuming. Another limitation is that this framework largely depends on the smoking information generated by the SHG, which has to be updated before it can be used to accurately predict properties of future lung cancer patient populations. We did not perform simulation by histology, which is another limitation. Moreover, the model did not consider the difference between the lung cancer risks in CPSI/NHS and SEER, while the previously estimation of parameters in carcinogenesis model was directly used. We cannot recalibrate carcinogenesis model since no smoking information was recorded in SEER.

## Conclusion

We proposed a model for predicting the natural disease progression and detection of lung cancer that relies on the following biologically and clinically reasonable assumptions: the hazard function of tumor progression is based on the activity of the tumor cells, which detach from the primary tumor and transfer to another part of the body, leading to metastases [13]. Thus, the metastasis process is related to the size of the primary tumor and the tumor growth rate (which is also related to the activity of the tumor cells). The detection of lung cancer in patients occurs as a result of competing detection of the primary tumor or nodal or distant metastasis. We used a TSCE model combined with the smoking history generator to reproduce the population with incipient tumors according to the yearly live birth number in the United States (Figure S1). We then applied our models of the tumor natural progression and its detection to re-create the lung cancer patient population at the time of diagnosis. Lung cancer data from SEER database collected between 2004 and 2008 were used to fit the lung cancer progression and detection model. The fitted model combined with a carcinogenesis model was used to reconstruct the distribution of age, gender, tumor size, and disease stage at diagnosis, and the results showed that the model accurately predicted gender and median age, and reasonably predicted the tumor size and disease stage distribution against independent data from the SEER database collected from 1988 to 1999. This model framework provides a platform for estimating the outcome of a strategy for the secondary prevention of lung cancer before it is applied in clinic.

## Supporting Information

Figure S1 **Yearly live birth number in US used in the simulation.** For years in which the number of live births was missing (between 1890 and 1908) we used the average number of live births between 1909 and 1928 (2,877,000).
(TIF)

Figure S2 **Comparison of U.S. population between simulated data and Census Bureau data; the simulated population deviates from the reality population after year 1984, since SHG could not generate new babies after 1984.** However, we expect only minor if any effect of that on the LC population, as lung cancer is very rare in young individuals.
(TIF)

Table S1 **Parameters of the response functions used in the TSCE model** [10] **.**
(DOCX)

Table S2 **Doubling time by stages and tumor size for the simulated LC population.**
(DOCX)

File S1 **Supporting text.**
(DOCX)
