# Supplementary figures and images for: Modeling the Natural History and Detection of Lung Cancer Based on Smoking Behavior
Source: PLoS One. 2014 Apr 4;9(4):e93430. doi: 10.1371/journal.pone.0093430 (PMC3976286; doi:10.1371/journal.pone.0093430)

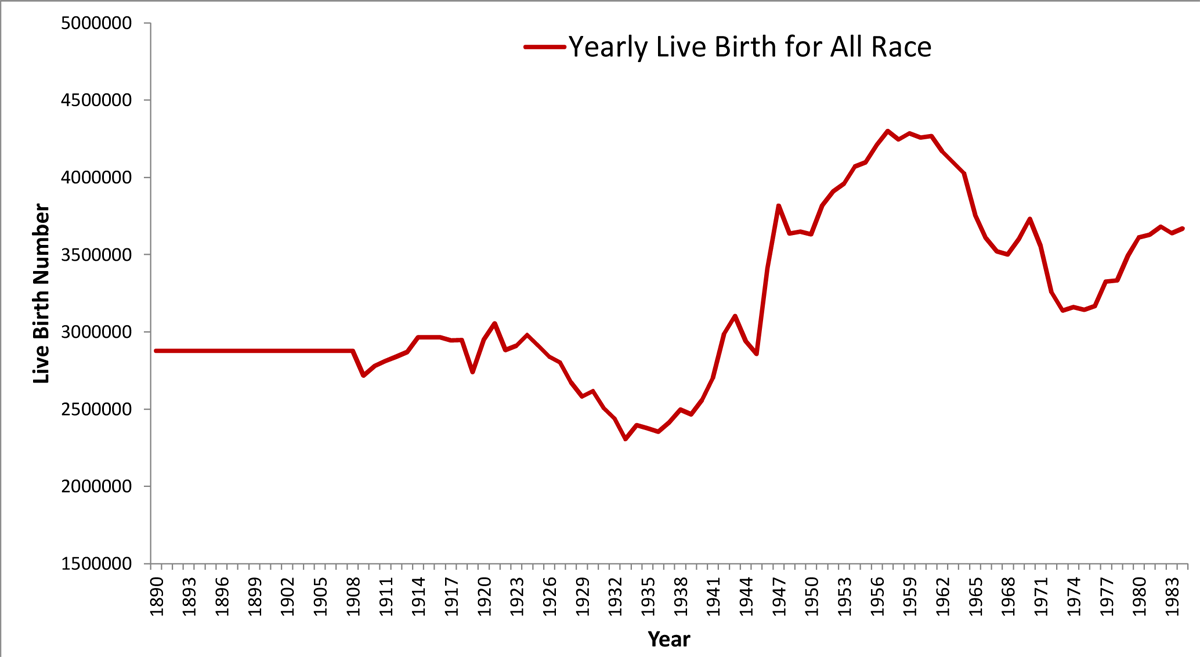

Supplement: Figure S1 — Yearly live birth number in US used in the simulation. For years in which the number of live births was missing (between 1890 and 1908) we used the average number of live births between 1909 and 1928 (2,877,000). (TIF) [file pone.0093430.s001.tif]

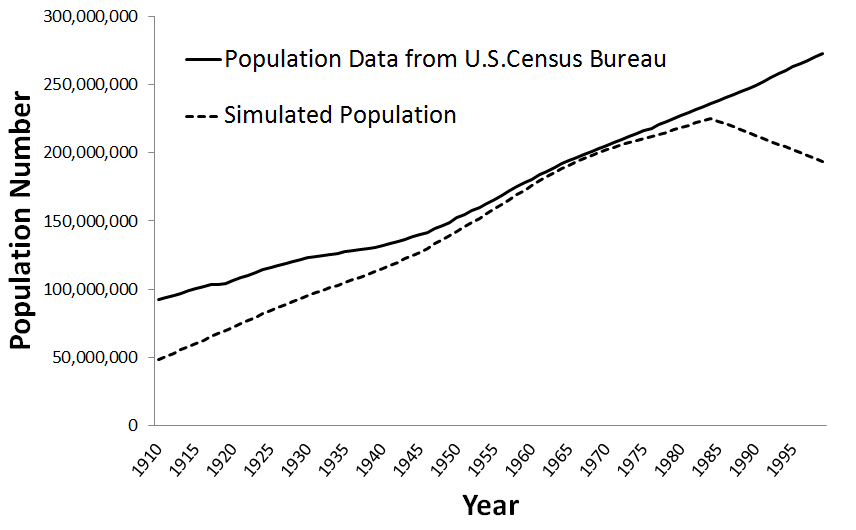

Supplement: Figure S2 — Comparison of U.S. population between simulated data and Census Bureau data; the simulated population deviates from the reality population after year 1984, since SHG could not generate new babies after 1984. However, we expect only minor if any effect of that on the LC population, as lung cancer is very rare in young individuals. (TIF) [file pone.0093430.s002.tif]
