# Supplementary material for: Modeling the Natural History and Detection of Lung Cancer Based on Smoking Behavior
Source: PLoS One. 2014 Apr 4;9(4):e93430. doi: 10.1371/journal.pone.0093430 (PMC3976286; doi:10.1371/journal.pone.0093430)
Supplement: Table S1 — Parameters of the response functions used in the TSCE model [10] . (DOCX) [file pone.0093430.s003.docx]

Table S1 Parameters of the response functions used in the TSCE model (Foy et al. 2011)

| Parameter* | $\alpha_{0}$ | $\gamma_{0}$ | $\nu_{0}X$ | $a_{1}$ | $a_{2}$ |
| --- | --- | --- | --- | --- | --- |
| Males (CPS-I) | 2.99 | 0.069 | 2.17 | 2.66 | 0.35 |
| Females (NHS) | 4.6 | 0.071 | 1.93 | 2.30 | 0.35 |

*Parameters for males and females were estimated using the data from Cancer Prevention Study I (CPS-I) and from Nurses’ Health Study (NHS), respectively.
