# Supplementary material for: Modeling the Natural History and Detection of Lung Cancer Based on Smoking Behavior
Source: PLoS One. 2014 Apr 4;9(4):e93430. doi: 10.1371/journal.pone.0093430 (PMC3976286; doi:10.1371/journal.pone.0093430)
Supplement: Table S2 — Doubling time by stages and tumor size for the simulated LC population. (DOCX) [file pone.0093430.s004.docx]

Table S2 Doubling time by stages and tumor size for the simulated LC population

|  |  |  |  | Tumor Size (cm) | | | | |
| --- | --- | --- | --- | --- | --- | --- | --- | --- |
|  | Stage Status | | | <0.5 | 0.5-1 | 1-2 | 2-3 | >3 |
| Doubling Time (days) | N0,M0 | Mean | | 87.98 | 80.95 | 78.66 | 77.77 | 48.28 |
|  |  | Median |  | 75.86 | 62.58 | 60.30 | 58.97 | 37.22 |
|  |  | Variance |  | 3194.09 | 2571.48 | 2617.51 | 2499.68 | 1338.48 |
|  |  | Std. Deviation | | 56.52 | 50.71 | 51.16 | 50.00 | 36.59 |
|  |  | Minimum |  | 18.71 | 18.09 | 17.52 | 17.18 | 12.87 |
|  |  | Maximum |  | 690.39 | 536.99 | 666.63 | 558.20 | 582.79 |
|  |  | Range |  | 671.67 | 518.90 | 649.10 | 541.02 | 569.91 |
|  |  | Interquartile Range | | 54.95 | 53.08 | 51.37 | 50.60 | 22.62 |
|  | N1,M0 | Mean |  | 62.17 | 59.27 | 59.26 | 60.77 | 48.00 |
|  |  | Median |  | 59.12 | 61.06 | 59.44 | 58.47 | 45.60 |
|  |  | Variance |  | 427.57 | 400.94 | 362.40 | 448.39 | 247.99 |
|  |  | Std. Deviation | | 20.68 | 20.02 | 19.04 | 21.18 | 15.75 |
|  |  | Minimum |  | 22.68 | 27.15 | 26.29 | 17.21 | 19.30 |
|  |  | Maximum |  | 381.36 | 380.33 | 434.59 | 534.34 | 539.51 |
|  |  | Range |  | 358.67 | 353.19 | 408.29 | 517.13 | 520.21 |
|  |  | Interquartile Range | | 15.30 | 13.78 | 23.38 | 22.94 | 18.16 |
|  | M1 | Mean |  | 117.32 | 114.54 | 112.79 | 111.48 | 75.97 |
|  |  | Median |  | 103.66 | 100.13 | 97.51 | 104.42 | 68.25 |
|  |  | Variance |  | 2657.77 | 2380.73 | 2280.73 | 2182.98 | 1240.29 |
|  |  | Std. Deviation | | 51.55 | 48.79 | 47.76 | 46.72 | 35.22 |
|  |  | Minimum |  | 28.18 | 36.28 | 26.82 | 25.98 | 19.32 |
|  |  | Maximum |  | 698.85 | 526.44 | 661.05 | 528.60 | 572.71 |
|  |  | Range |  | 670.67 | 490.16 | 634.23 | 502.63 | 553.39 |
|  |  | Interquartile Range | | 50.71 | 49.27 | 48.13 | 46.79 | 39.52 |
