# Supplementary material for: Modeling the Natural History and Detection of Lung Cancer Based on Smoking Behavior
Source: PLoS One. 2014 Apr 4;9(4):e93430. doi: 10.1371/journal.pone.0093430 (PMC3976286; doi:10.1371/journal.pone.0093430)
Supplement: File S1 — Supporting text. (DOCX) [file pone.0093430.s005.docx]

## *Supplementary file*

## *1.1. Carcinogenesis modeling*

The standard form of this two-stage model involves four parameters (*ν(t), α(t), β(t),* and *µ(t)* ). This model assumes that a normal cell mutates to an initiated cell according to a Poisson process with intensity *ν(t)*, where *t* denotes age. The initiated cell can then either replicate or die according to a birth-death process with the parameters *α(t)* and *β(t)*. Each initiated cell can also mutate into a malignant cell according to a Poisson process with the parameter *µ(t)*. Once a malignant cell is formed, the assumption is made that it will proliferate into a malignant tumor, resulting in lung cancer with a probability of one.

A smoking-based modification of the TSCE model relating smoking intensity measured in packs per day (ppd) to the parameters of the TSCE model through response functions was shown in the following.

$$X={10}^{7}$$

$$v\left( t \right)= v_{0}X(1+a_{1}\times\sqrt{ppd})$$

$$\mu\left( t \right)= v_{0}(1+a_{1}\times\sqrt{ppd})$$

$$\alpha\left( t \right)= \alpha_{0}(1+a_{2}\times\sqrt{ppd})$$

$\gamma\left( t \right)= {\alpha\left( t \right)-\beta\left( t \right)-\mu\left( t \right)=\gamma}_{0}(1+a_{2}\times\sqrt{ppd})$

Here *ppd* stands for packs per day of smoking.

Smoking duration was incorporated to produce a more specific Survival function of the age at tumor initiation for individual never, current and former smokers. ([Foy et al. 2011c](#_ENREF_13), [Foy et al. 2011b](#_ENREF_12), [Foy et al. 2011a](#_ENREF_11)).

## *2.1. Simulation Process*

For each simulated person, we randomly assigned male or female gender with the probability of 50%, which was used in the SHG to generate smoking information. We used the age at death due to causes other than lung cancer, *A_d_*, generated by the SHG (version 5.2.1) as one of the inputs to the carcinogenesis model. Technically, we generated a random variable R_i_ from a uniform distribution on (0, 1). If R_i_ was larger than the survival function of the carcinogenesis model at the age *A_d_*, then the person developed lung cancer in his or her lifetime. Otherwise, the person died from other causes and was excluded from the pool. If the person did not die before the year 2000, we used their attained age at year 2000 as the censoring age, consistent with the SHG using the year 2000 as a cut-off point for the vital status observation.

To calculate the age of the patient at tumor initiation, we used *R_i_* as input for the inverse carcinogenesis model survival function to generate *T*_0_ (the age at tumor initiation). We could then calculate the primary tumor size at any time (including the time at death of other causes) using the tumor-growth model with an assigned growth rate. For example, the size of tumor at death of other causes equals $S_{d}=e^{\lambda(A_{d}-T_{0})}$, where growth rate λ is generated from a gamma distribution.

To determine whether a patient with lung cancer developed nodal or distant metastases during his/her lifetime, another random number, *R_m_*(0≤*R_m_*≤1), was generated. If $R_{m}\leq F_{n}(S_{d})$ or $R_{m}\leq F_{m}(S_{d})$, then the patient developed nodal or distant metastases before the time at death of other causes. The primary tumor size at the initiation of nodal and distant metastases, *S_ni_* or *S_mi_*, respectively, is

$$S_{ni}={F_{n}}^{-1}\left( R_{m} \right) S_{mi}={F_{m}}^{-1}(R_{m})$$

According to the tumor growth model and the assumption that metastases grow faster than the primary tumor, we were able to generate the sizes of the primary and metastases, nodal or distant, at any point in time. Thus, we can apply the detection method in the detection model to detect the primary tumor and nodal and distant metastases separately and competitively.

The model traces the sizes of the primary tumor and nodal and distant metastases. Again, we generate uniform (0, 1) random numbers *R_di_*, (i=0, 1, 2), and if the condition

$${[R}_{d0}\leq D_{p}(s_{p})]or [R_{d1}\leq D_{n}(s_{n})]or [R_{d2}\leq D_{m}(s_{m})]$$

is satisfied, then the person is considered detected and is included in the detected population. The sizes of the primary tumor and nodal and distant metastases at detection are denoted as $s_{p}, s_{n}, s_{m}$. If not detected, the person survives another time interval and the detection model is repeated until his/her death. Thus, for each person simulated, we obtain age, smoking status, gender, tumor size, and disease stage information at the time of diagnosis. We then repeat the simulations for each live person born in each year from 1890 to 1984 (Supplementary Figure 1) to obtain the simulated lung cancer patient population for each year. We estimated the nine parameters (*ξ,* $\mu_{n}$*,* $\mu_{m}$, $\lambda(K, \theta)$, η, *W*_0_, *W*_1_, *W*_2_) in the tumor-growth and metastasis models using the data in the SEER database from 2004 to 2008. Because the TNM staging information was not available in the SEER database before 2004, the joint distributions of tumor size and disease stage (N0,M0: no metastases; Nx,M0: only nodal metastases; and M1: distant metastases) from 1995 to 1999 were chosen as the output of the simulation.

## *2.2. Estimating the 95% confidential interval of model parameters*

We independently estimated the 95% confidence interval for each parameter. We randomly assigned an initial value (within a reasonable range) of one parameter and applied the Nelder-Mead method to achieve the best fitted value of that parameter in the model. We repeated the procedure 50 times for each parameter and calculated their corresponding 95% confidence intervals.
